# Connection between right-to-left shunt and photosensitivity: a community-based cross-sectional study

**DOI:** 10.3389/fneur.2023.1177879

**Published:** 2023-04-27

**Authors:** Bosi Dong, Shuming Ji, Yajiao Li, Hua Li, Ruiqi Yang, Na Yang, Zhu Liu, Chenxing Zhu, Hui Wang, Yusha Tang, Anjiao Peng, Lei Chen

**Affiliations:** ^1^Department of Neurology, West China Hospital, Sichuan University, Chengdu, China; ^2^Department of Clinical Research Management, West China Hospital, Sichuan University, Chengdu, China; ^3^Department of Cardiology, West China Hospital, Sichuan University, Chengdu, China

**Keywords:** right-to-left shunt, photosensitivity, migraine, community-based study, cross-sectional study

## Abstract

**Background:**

Hypersensitivity to light is a common symptom associated with dysfunction of the occipital region. Earlier studies also suggested that clinically significant right-to-left shunt (RLS) could increase occipital cortical excitability associated with the occurrence of migraine. The aim of this study was to investigate the relationship between RLS and photosensitivity.

**Methods:**

This cross-sectional observational study included the residents aged 18–55 years living in the Mianzhu community between November 2021 and October 2022. Photosensitivity was evaluated using the Photosensitivity Assessment Questionnaire along with baseline clinical data through face-to-face interviews. After the interviews, contrast-transthoracic echocardiography (cTTE) was performed to detect RLS. Inverse probability weighting (IPW) was used to reduce selection bias. Photosensitivity score was compared between individuals with and without significant RLS using multivariable linear regression based on IPW.

**Results:**

A total of 829 participants containing 759 healthy controls and 70 migraineurs were finally included in the analysis. Multivariable linear regression analysis showed that migraine (β = 0.422; 95% CI: 0.086–0.759; *p* = 0.014) and clinically significant RLS (β = 1.115; 95% CI: 0.760–1.470; *p* < 0.001) were related to higher photosensitivity score. Subgroup analysis revealed that clinically significant RLS had a positive effect on hypersensitivity to light in the healthy population (β = 0.763; 95% CI: 0.332–1.195; *p* < 0.001) or migraineurs (β = 1.459; 95% CI: 0.271–2.647; *p* = 0.010). There was also a significant interaction between RLS and migraine for the association with photophobia (*p*_interaction_ = 0.009).

**Conclusion:**

RLS is associated with photosensitivity independently and might exacerbate photophobia in migraineurs. Future studies with RLS closure are needed to validate the findings.

**Trial registration:**

This study was registered at the Chinese Clinical Trial Register, *Natural Population Cohort Study of West China Hospital of Sichuan University*, ID: ChiCTR1900024623, URL: https://www.chictr.org.cn/showproj.html?proj=40590.

## 1. Introduction

Photophobia, a high sensitivity to light (photosensitivity), is a common sensory disturbance seen in several neurological conditions, especially migraine which is the third most prevalent disease worldwide (1, 2). There are ~70–80% of migraineurs experiencing photophobia (3). Current analyses show that photosensitivity of migraine is the most bothersome non-headache symptom, and it still exists during the interictal period (4). It has also been reported that history of abuse, trauma, meningitis, and intracranial tumors are associated with photosensitivity (5, 6). However, these factors cannot fully account for the development of photophobia, and the mechanism of interictal photosensitivity in migraine remains unclear.

Meanwhile, the incidence of a right-to-left shunt (RLS) is ~50–60% in migraineurs, which is similar to that of photophobia (7, 8). RLS allows micro-emboli, air emboli, and partial unoxygenated venous blood to enter the systemic circulation directly, which can trigger cortical spreading depression (CSD) (9, 10). CSD is a phenomenon that extends from the initial site of excessive neuronal excitability to the surrounding tissues and further causes the continuous inhibition of cortical electrical activity (11). CSD, which often initially occurs in the visual cortex, provides the most likely explanation for visual symptoms in migraine (12). The occipital cortex and connected regions are also hyperexcitable in subjects with photosensitivity, including migraine with interictal photosensitivity, idiopathic generalized epilepsy, anxiety, and depression (13–15). Moreover, the induction of CSD can also increase the activity of trigeminovascular neurons, contributing to the formation of visual phenomena (16). Photophobia is also associated with dysfunction of the trigeminovascular pathway which could alter the responsiveness to visual stimuli (17). Basic studies have also verified that the performance of photophobia may be due to an enhanced interaction of trigeminal and visual inputs through the neuronal pannexin-1 channel opened by the spread of CSD (18). Furthermore, RLS could cause circulation disorders involved with autonomic dysfunction which is also associated with photosensitivity (19). The clinical evidence suggests that RLS-induced embolic events might be linked to the clinical manifestation of cutaneous vasculitis and photosensitivity (20). Nevertheless, the relationship between RLS and photosensitivity has never been explored directly. Thus, we designed a community-based cross-sectional study with the primary aim to investigate the correlation between clinically significant RLS and photosensitivity.

## 2. Materials and methods

### 2.1. Participants and data collection

This cross-sectional study was part of the ongoing community-based Mianzhu study, a cohort study designed to investigate the risk factors and burden of chronic diseases in Chinese community-dwelling adults. All inhabitants over the age of 18 who had lived in communities for more than 6 months in Mianzhu (a rural district located 50 miles from urban area of Chengdu) were invited by text message recruitment letters and banner advertisements. From November 2021 to October 2022, 1,533 individuals aged 18–55 years participated, and their standard baseline assessments were undertaken. All participants were also invited for contrast-transthoracic echocardiography (cTTE) examination and photosensitivity assessment. Participant exclusion criteria for this analysis were as follows: (1) self-reported history of ophthalmological conditions; (2) self-reported history of heart disease except RLS, distinct respiratory, neurological or psychiatric diseases, and/or use of psychotropic medication (present and past); (3) 9-item Patient Health Questionnaire (PHQ9) score > or = 15 (21); (4) 7-item General Anxiety Disorder Scale (GAD7) score > or = 10 (22); (5) cTTE not completed; and (6) unwillingness to take cTTE or photosensitivity assessment.

All baseline data were collected from clinician-entered information *via* face-to-face interviews based on the standard structured questionnaire. The following information was assessed: age, gender, educational level, body mass index (BMI), smoking, alcohol drinking, and regular coffee intake. Educational levels were divided into low (≤ 9 years) and high (more than 9 years). Smoking was defined as having at least one cigarette per day for more than 1 year. Alcohol drinking was defined as having at least one drink a week for more than half a year. Regular coffee was defined as having coffee at least three times a week for more than half a year. Migraine was diagnosed by two specialists independently based on the guidelines of the International Classification of Headache Disorders (ICHD-3 beta) (23). As for migraineurs, approximate years lived with headache and headache frequency (calculated by self-reported headache days per month on average over the last 3 months) were recorded (6). The 6-item Headache Impact Test (HIT6) rating the severity of the underlying migraine disorder (24) was also assessed.

All the participants provided written informed consent. This study complied with the Declaration of Helsinki. The study was approved by the Ethics Committee of Sichuan University (No. 2020145) and registered at the Chinese Clinical Trial Register (ChiCTR1900024623).

### 2.2. Photosensitivity assessment

Photosensitivity was measured using the Photosensitivity Assessment Questionnaire, which could not only specifically address changes in light sensitivity caused by headache but also assess general photosensitivity (25). The thirteen items regarding photophobia and the 10 items regarding the photophilia score ranging from 0 to 1 were separately calculated from responses. The mean photophobia score and the photophilia score were reported at 4.99 (SD 2.64) and 4.15 (SD 1.98) in healthy people, respectively (25). The questionnaire was developed and validated in an Italian population, while it had never been used in studies with the Chinese population. Translation for the scales was performed by two certified translation specialists. To ensure that the versions in Chinese were the same as those in Italian, a back-to-back translation strategy was used. After translation, we also adapted the scale to account for Chinese cultural characteristics. We had multiple face-to-face meetings to discuss the rating and wording of statements for finalizing the draft scale. After we agreed on the content validity of the statements in the Photosensitivity Assessment Questionnaire, the scale was released to the participants (see Supplementary Table 1). For migraineurs, all of them completed the questionnaire in the interictal state. All obtained data were used to evaluate the reliability and validity of the Photosensitivity Assessment Questionnaire. Raw Cronbach's alpha of our Photosensitivity Assessment Questionnaire was 0.816, and the standardized alpha was 0.818 for this study. The overall Comparative Fit Index (CIF) was good at 0.768. Only the photophobia score of the Photosensitivity Assessment Questionnaire refers to “photosensitivity” and has been analyzed in previous studies (6, 13); thus, we evaluated only questions relating to photophobia, which we will continue to refer to within this article as “photosensitivity”.

### 2.3. RLS screening

Participants were invited to undergo cTTE after collecting clinical characteristics and finishing the Photosensitivity Assessment Questionnaire. First, transthoracic echocardiography (TTE) was performed using 1–5 MHz or 3–8 MHz multiplane transducers in a Philips IE33 for each participant. In this step, participants would be excluded if other cardiac diseases were identified by two experienced sonographers. Next, sonographers analyzed RLS by cTTE. A contrast medium was made by shaking the solution mixing 1 ml of blood and 1 ml of air with 8 ml of saline and then injected into the antecubital veins for increased sensitivity (26). RLS was assessed at rest, during a Valsalva maneuver and coughing, and the highest number of microbubbles in the left atrium observed was recorded, either spontaneously or after provocative maneuvers. For semiquantitative analysis, the degree of RLS assessed by cTTE was divided into four groups based on the International Consensus Criteria as follows: Grade 0, no occurrence of microbubbles; Grade 1, 1–10 microbubbles; Grade 2, 10–30 microbubbles; and Grade 3, > 30 microbubbles or left atrium nearly filled with microbubbles or left atrial opacity (27). Grades 1 and 0 were considered as insignificant or no RLS group, and Grades 2 and 3 were considered as clinically significant shunt group.

### 2.4. Statistical analysis

Categorical variables were described as frequencies and percentages, and continuous variables were described as mean ± SD. For comparison between healthy controls and migraineurs, the χ^2^ test (or Fisher's exact test when any expected cell count was <5 for a 2 × 2 table) was used for categorical variables, and the independent Student's *t*-test was used for continuous variables. Inverse probability weighting (IPW) was used to adjust for differences in baseline characteristics of healthy controls and migraineurs. A standardized mean difference (SMD) after IPW <0.1 was acceptable. Significance was tested using the regression models weighted by the same weights. Multivariate linear regression models were used to determine whether RLS was independently associated with photophobia in all participants and subgroups of healthy controls and migraineurs, respectively. We also used the function “visreg” from the visreg package to visualize the contrast plot of the RLS and migraine interaction effects in photophobia. A *p*-value of < 0.05 was considered to be statistically significant. All statistical analyses were conducted in R 4.0.1.

## 3. Results

### 3.1. Basic clinical data

**Figure 1:**
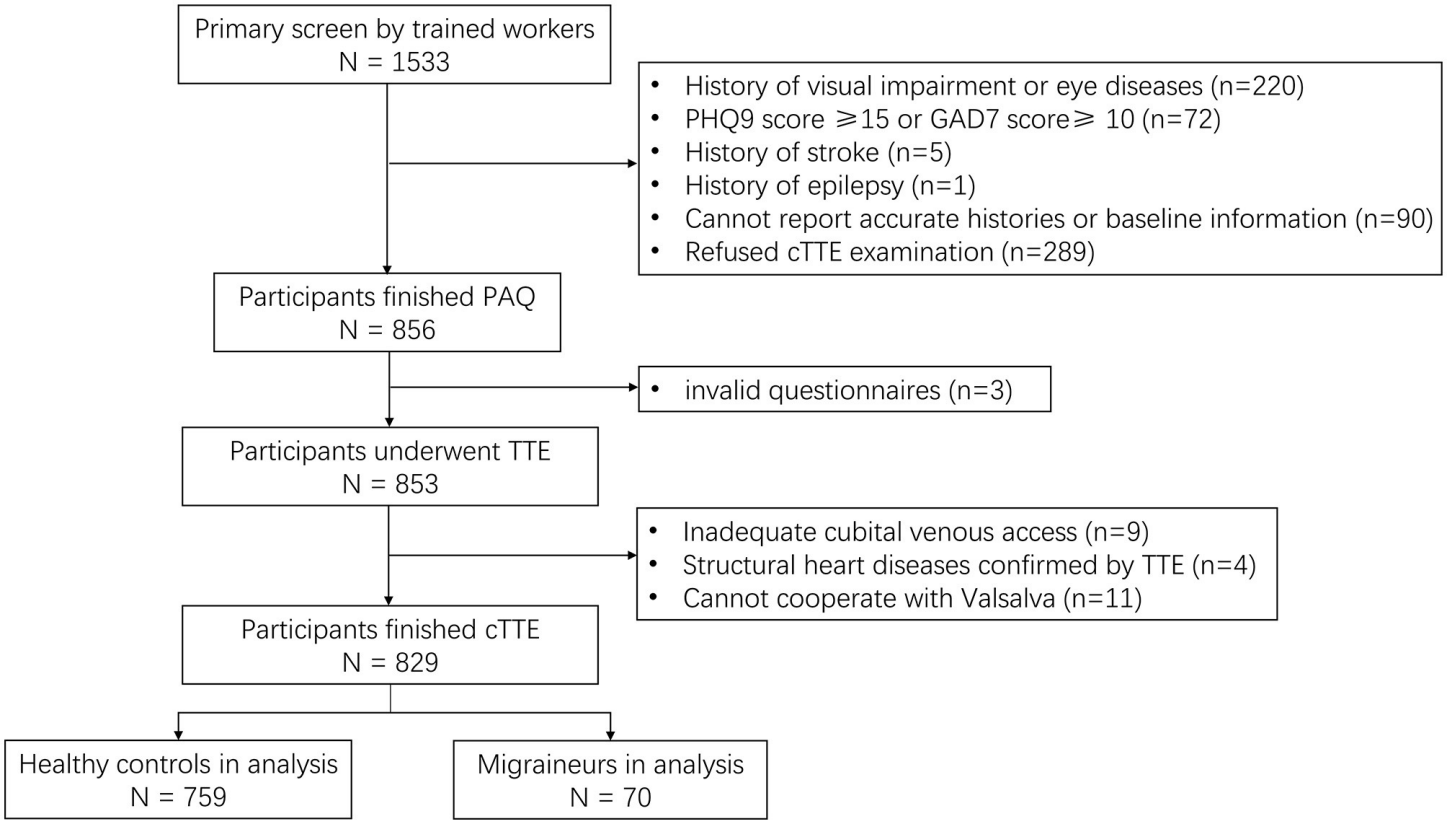
Flowchart of patient recruitment. TTE, transthoracic echocardiography; cTTE, contrast medium transthoracic echocardiography; PAQ, Photosensitivity Assessment Questionnaire; PHQ, Patient Health Questionnaire; GAD, General Anxiety Disorder.

**Figure 2:**
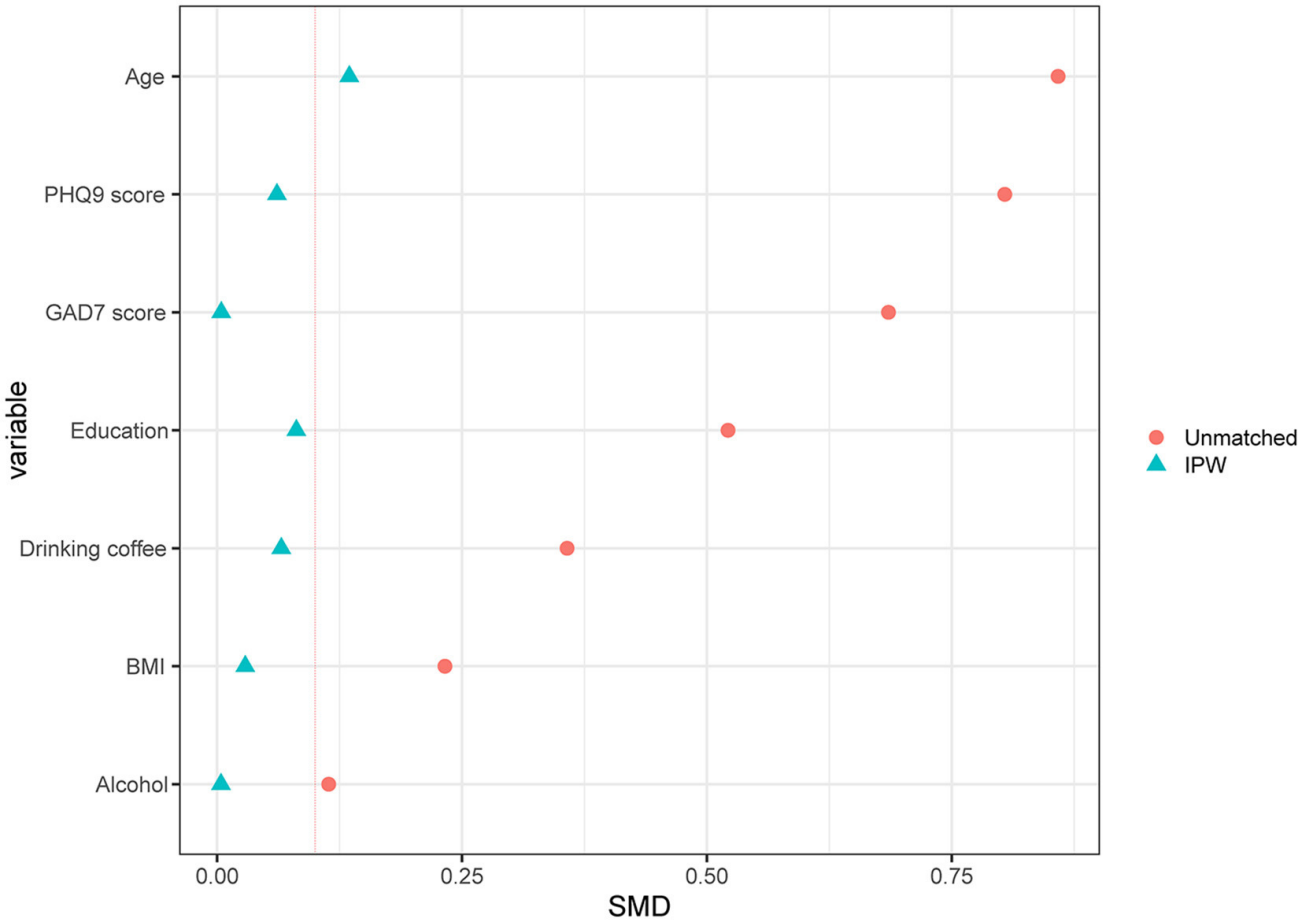
Standardized differences shown for each confounder variable of RLS. The X-axis represents the standardized differences value, and the Y-axis represents baseline variables. The red vertical line signifies the standardized differences cutoffs of >0.1. RLS, right-to-left shunt; IPW, inverse probability weighted; SMD, standardized mean difference.

Finally, 829 participants were included in the analysis, including 759 healthy controls and 70 migraineurs. The flowchart of the study inclusion process is shown in Figure 1. The mean age (SD) of the participants was 47.81 (6.61) years, and the ratio of female participants was 80.1% (664/829). Clinically significant RLS was observed in 25.3% of all participants (210/829), and none of the participants had atrial septal aneurysm. As for migraineurs, 12.86% of them (9/70) were diagnosed with chronic migraine according to ICHD-3 beta, and only one patient had migraine with medication overuse (characteristics of different types of migraine are shown in Supplementary Table 2). The characteristics of the migraineurs and healthy controls are presented in Table 1. Migraineurs had a higher incidence of significant RLS (37.1% vs. 24.2%, *p* = 0.026) and a higher photosensitivity score than healthy controls (5.39 vs. 4.57, *p* = 0.013). To account for selection bias, observed differences in baseline characteristics between the healthy controls and migraineurs were controlled with IPW-adjusted analyses (Figure 2). After the inverse probability of weighting, the SMD of age was 0.135, and the absolute SMD of other variables was all <0.1, indicating that the weighted cohorts were comparable. The results of all remaining analyses were based on IPW-adjusted analyses.

**Table 1:** Unweighted demographic characteristics of migraineurs and healthy controls.

	**Healthy controls (*n* = 759)**	**Migraineurs (*n* = 70)**	***p***
Gender (male, %)	152 (20.0)	13 (18.6)	0.892^†^
Age (y, mean ± SD)	48.43 (5.74)	41.11 (10.60)	**<0.001** ^‡^
Education (≤ 9 y, %)	584 (76.9)	37 (52.9)	**<0.001** ^†^
BMI (mean ± SD)	23.73 (3.15)	22.92 (3.80)	**0.043** ^‡^
Smoke (*n*, %)	93 (12.3)	8 (11.4)	0.991^†^
Alcohol (*n*, %)	211 (27.8)	16 (22.9)	0.455^†^
Coffee (*n*, %)	67 (8.8)	15 (21.4)	**0.002** ^†^
PHQ9 score (mean ± SD)	0.75 (1.38)	2.77 (3.28)	**<0.001** ^‡^
GAD7 score (mean ± SD)	0.74 (1.55)	2.93 (4.23)	**<0.001** ^‡^
Significant shunt (*n*, %)	184 (24.2)	26 (37.1)	**0.026** ^†^
Photosensitivity score (mean ± SD)	4.57 (2.63)	5.39 (2.56)	**0.013** ^‡^

Bold indicates significant differences at *p* < 0.05.

† χ^2^ test.

‡ Student's *t*-test.

### 3.2. Association of clinical variables with photosensitivity and multivariable adjustment

We noticed that all the participants with significant RLS had a higher score of photosensitivity (5.21 ± 2.54) compared to those without significant RLS (4.45 ± 2.64, *p* < 0.001) (Table 2). Univariate analysis of photosensitivity score revealed that older age (β = 0.041; *p* < 0.01), larger weight (β = 0.125; *p* < 0.001), lower education (β = −1.151; *p* < 0.001), migraine (β = 0.711; *p* < 0.001), and clinically significant RLS (β = 1.362; *p* < 0.001) were associated with greater photosensitivity (Supplementary Table 3). After controlling for the effects of all baseline variates, migraine (β = 0.422; 95% CI: 0.086–0.759; *p* = 0.014) and clinically significant RLS (β = 1.115; 95% CI: 0.760–1.470; *p* < 0.001) were still independently related to greater photosensitivity in the multivariate linear regression (Table 3). In the subgroup of healthy controls, individuals with clinically significant RLS had a higher photosensitivity than those without significant RLS (5.05 vs. 4.42, *p* = 0.004) (Table 2), and RLS was still associated with higher photosensitivity score after considering all variates (β = 0.763; 95% CI: 0.332–1.195; *p* < 0.001) (Table 3). As for migraineurs, those with significant shunt also had a higher photosensitivity score than those with insignificant or no shunt (4.82 vs. 6.35, *p* = 0.015) (Table 2). The association of greater photosensitivity and significant shunt remained significant in migraineurs after controlling baseline variates (β = 1.459; 95% CI: 0.271–2.647; *p* = 0.017). We furtherly controlled the effects of headache characteristics, and it was also evident that RLS was significantly correlated with photosensitivity independently (β = 1.560; 95% CI: 0.373–2.746; *p* = 0.010). There was an insignificant relationship between photosensitivity and years lived with headache, headache frequency, aura, chronic migraine, or HIT6 in multivariate linear regression analysis (Table 3).

**Table 2:** High photosensitivity score in participants with significant RLS in the group of healthy controls or group of migraineurs.

	**Health controls (*****n*** = **759)**			**Migraineurs (*****n*** = **70)**		
	**Insignificant or no shunt (*****n*** = **575)**	**Significant shunt (*****n*** = **184)**	***p***	**Insignificant or no shunt (*****n*** = **44)**	**Significant shunt (*****n*** = **26)**	***p***
Gender (male, %)	123 (21.4)	29 (15.8)	**0.120** ^†^	9 (20.5)	4 (15.4)	0.834^§^
Age (y, mean ± SD)	48.46 (5.79)	48.34 (5.58)	0.810^‡^	40.52 (9.84)	42.12 (11.91)	0.547^‡^
Education (≤ 9 y, %)	446 (77.6)	138 (75.0)	0.536^†^	21 (47.7)	16 (61.5)	0.384^†^
BMI (mean ± SD)	23.89 (3.20)	23.23 (2.97)	**0.014** ^‡^	22.36 (3.37)	23.86 (4.34)	0.110^‡^
Smoke (n, %)	72 (12.5)	21 (11.4)	0.787^†^	7 (15.9)	1 (3.8)	0.253^§^
Alcohol (n, %)	163 (28.3)	48 (26.1)	0.616^†^	10 (22.7)	6 (23.1)	1.000^†^
Coffee (n, %)	55 (9.6)	12 (6.5)	0.264^†^	9 (20.5)	6 (23.1)	1.000^†^
PHQ9 score (mean ± SD)	0.69 (1.34)	0.93 (1.50)	**0.037** ^‡^	3.34 (3.68)	1.81 (2.17)	0.058^‡^
GAD7 score (mean ± SD)	0.73 (1.53)	0.80 (1.62)	0.576^‡^	3.14 (4.36)	2.58 (4.07)	0.597^‡^
Photosensitivity score (mean ± SD)	4.42 (2.65)	5.05 (2.53)	**0.004** ^‡^	4.82 (2.55)	6.35 (2.31)	**0.015** ^‡^
**Migraine characteristics**						
Aura (*n*, %)	/	/	/	1 (2.3)	3 (11.5)	0.280^§^
Headache frequency (mean ± SD)	/	/	/	5.18 (7.16)	3.33 (6.34)	0.279^‡^
Age at onset (y, mean ± SD)	/	/	/	23.66 (8.64)	23.23 (8.80)	0.843^‡^
Years lived with headache (y, mean ± SD)	/	/	/	16.86 (9.52)	18.88 (11.88)	0.437^‡^
Chronic migraine (n, %)	/	/	/	7 (15.9)	2 (7.7)	0.533^†^
HIT6 (mean ± SD)	/	/	/	58.48 (11.58)	50.65 (11.71)	**0.008** ^‡^

Bold indicates significant differences at *p* < 0.05.

† χ^2^ test.

‡ Student's *t*-test.

§ Fisher's exact test.

**Table 3:** Multivariate linear regression models of photosensitivity scores in healthy controls and migraineurs.

	**Coefficient (95% CI) of multivariate models**		
	**All participants**	**Healthy controls**	**Migraineurs**
Significant shunt	1.115^***^ (0.760, 1.470)	0.763^***^ (0.332, 1.195)	1.459^*^ (0.271, 2.647)
Migraine	0.422^*^ (0.086, 0.759)	/	/
Male	−0.082 (−0.650, 0.486)	−0.334 (−0.920, 0.253)	0.552 (−1.687, 2.790)
Age	0.017 (−0.012, 0.045)	0.039 (0.005, 0.072)	0.009 (−0.095, 0.113)
BMI	0.076^*^ (0.017, 0.134)	0.093^**^ (0.033, 0.153)	0.031 (−0.193, 0.256)
Education	−0.950^***^ (−1.395, −0.505)	−0.500^*^ (−0.980, −0.019)	−1.495 (−3.083, 0.093)
Coffee	0.454 (−0.089, 0.997)	−0.042 (−0.678, 0.594)	1.041 (−0.756, 2.838)
Smoke	0.060 (−0.662, 0.782)	−0.775 (−1.477, −0.073)	2.163 (−0.943, 5.270)
Alcohol	0.153 (−0.254, 0.560)	−0.117 (−0.565, 0.332)	0.474 (−1.000, 1.949)
PHQ9 score	0.026 (−0.086, 0.137)	0.060 (−0.080, 0.200)	−0.025 (−0.385, 0.336)
GAD7 score	0.083 (−0.019, 0.186)	−0.068 (−0.193, 0.058)	0.184 (−0.160, 0.527)
**Migraine characteristics**			
Significant shunt	/	/	1.560^**^ (0.373, 2.746)
Age at onset	/	/	0.041 (−0.058, 0.140)
Years lived with headache	/	/	−0.007 (−0.093, 0.079)
Aura	/	/	0.138 (−1.865, 2.141)
Headache frequency	/	/	0.087 (−0.194, 0.368)
Chronic migraine	/	/	−3.020 (−8.106, 2.067)
HIT6	/	/	−0.001 (−0.055, 0.052)

^*^p < 0.05, ^**^p < 0.01, ^***^p < 0.001.

### 3.3. Association of RLS, migraine, and photosensitivity

**Figure 3:**
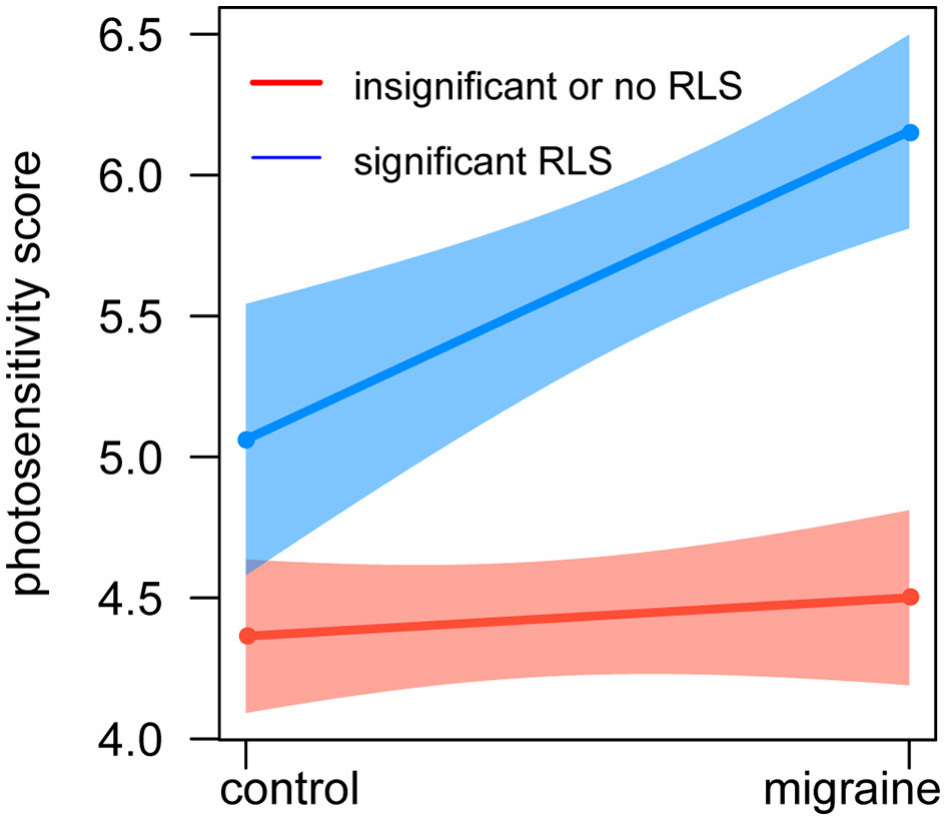
Relationship between photosensitivity, RLS, and migraine. The red line represents insignificant or no shunt, the blue line represents significant shunt, and the whiskers show a 95% CI. The X-axis represents the groups of healthy control and migraine, and the Y-axis represents the photosensitivity score. There was an interaction between shunt and migraine for the association with greater photosensitivity. CI, confidence interval.

Additionally, an interaction term between significant RLS and migraine was also created and entered as a factor of photosensitivity score in the regression model. There was a significant interaction between clinically significant RLS and migraine for the association with greater photosensitivity (β_interaction_ = 0.957; 95% CI: 0.234–1.678; *p*_interaction_ = 0.009), and Figure 3 shows that RLS could increase photosensitivity scores in migraine.

## 4. Discussion

In this study, participants from the community were assessed for photosensitivity by the adjusted and validated Photosensitivity Assessment Questionnaire, as well as for RLS by the cTTE. The results showed that (1) individuals with clinically significant RLS had a higher photosensitivity score compared with those without RLS; (2) RLS was an independent factor associated with hypersensitivity to light; and (3) there was an interaction between RLS and migraine for the association with photosensitivity, suggesting that RLS might worsen photophobia in migraineurs.

Photophobia is a light-induced phenomenon with underlying pathophysiologic mechanisms in visual, trigeminal, and autonomic systems so far; however, the factors of this increased photosensitivity are incompletely understood, especially in non-migraine (28). In this study, we tested the relationship between RLS and photosensitivity directly and found higher photosensitivity in the population with significant RLS and that RLS was an independent risk factor of hypersensitivity to light. The connection between RLS and photosensitivity may have several explanations. The generation of CSD induced by RLS was reported to be involved with the occipital cortex, which might underlie many visual symptoms, such as long-term contrast discrimination, visual evoked potential prolongation, and visual field sensitivity (29–31). Hypersensitivity to light was also associated with cortical hypersensitivity within the visual cortex (32). Animal experimental research verified that air micro-emboli could trigger CSD in the posterior region of the mice's brain and induce obvious photophobia in mice (10, 33). Moreover, functional magnetic resonance imaging (fMRI) studies described the activation of the trigeminothalamic pathway including central changes in individuals with high photosensitivity (34). There was also evidence that RLS-induced CSD mediated activation of trigeminal primary afferents (35). In addition to CSD, several studies suggested that RLS had an impact on hemodynamics in the anterior and posterior circulation through frequent transit of micro-emboli or vasoactive substances bypassing the deactivating pulmonary filters (36, 37). The posterior microcirculation abnormalities were related to the dysfunction of the autonomic system with the manifestation of photophobia (38, 39). In addition, prior clinical surveys proved that paradoxical embolism induced by RLS could damage both cerebral and peripheral microcirculation, leading to an alteration of serotonin, pro-inflammatory bradykinin, or neurotensin metabolism (40). These metabolic impairments also had an adverse effect on autonomic function, causing disturbances in the response to visual stimuli (41, 42).

Additionally, our results were consistent with the previous findings that the prevalence of migraine in the Asian population was nearly 9% and photophobia was a common interictal characteristic of migraine (43, 44). Our finding also showed that the interaction between RLS and migraine was significant in photophobia, suggesting that RLS could aggravate photosensitivity in migraine. On the one hand, interictal photophobia may be a symptom of a structural and functional disorder of the posterior head in migraine. Prior clinical studies had established a link between migraine and increased interictal activation of visual processing brain regions in response to a visual stimulus (45). Migraineurs performed hyperexcitability of the visual cortex with a wider photo-responsive area during interictal periods in fMRI studies (46). Transcranial static magnetic field stimulation of the visual cortex and greater occipital nerve block could improve photophobia in migraine (47, 48). On the other hand, RLS could be one of causal factors for the development of migraine, probably due to RLS-induced CSD in the calcarine cortex. Our result was in concordance with a higher incidence of RLS in migraineurs (49). Clinical research found that the paradoxical air micro-embolism bypass RLS could develop CSD or CSD-like bioelectrical abnormalities in occipital lobes and then occasionally induce headache (50). In addition, neuroimaging evidence showed micro-embolic origin located in the posterior vascular border zone territory in young migraineurs (51), and researchers concluded that the presence of RLS might be responsible for the development of migraine by affecting the integrity of the white matter in posterior circulation and the brain function of the posterior region (52). Thus, we speculated that RLS might contribute to cerebral hemodynamic disorders, particularly in the posterior head, which would further aggravate the symptom of hypersensitivity to light in migraineurs. In previous studies, the reduction of visual stimulation was suggested to be a useful tool for reducing migraine attacks (53). It has also been reported that there was a reduction in the frequency and severity of migraine attacks after RLS closure in migraineurs (54). Nevertheless, some negative studies proposed that patient selection for RLS closure should be careful, and indications for cessation of RLS in migraine remained challenging (55). We therefore considered that photosensitivity score needed to be evaluated in future RLS closure trials to validate the association and that photosensitivity might also be a factor for future evaluation of RLS closure in migraineurs.

Our study also had limitations. The sample size of migraine in the community was small, which probably led to the lack of significant correlation between photophobia and migraine with aura. The results should be further investigated in future large migraine case–control studies. Meanwhile, there was a mismatch in age between migraineurs and health controls, which might be due to the large age span and small sample size of the recruited migraineurs. Though the effect size of SMD of age <0.2 was considered negligible, care is still needed when generalizing the results. In addition, there was a relatively small sample size of male; however, migraine affects more women than men (56). In addition, ophthalmic and neurological diseases were mainly self-reported and rarely confirmed by imaging evaluations. The cross-sectional study design was inherently subject to bias, especially recall bias, as well as the effect of unmeasured confounders.

In conclusion, our study presents that RLS is associated with photosensitivity independently and that RLS could aggravate photosensitivity in migraineurs. Further investigation of a possible mechanistic link between RLS and photosensitivity is warranted, which might help in evaluating RLS closure operation.

## Data availability statement

The raw data supporting the conclusions of this article will be made available by the authors, without undue reservation.

## Ethics statement

The studies involving human participants were reviewed and approved by Ethics Committee of Sichuan University. The patients/participants provided their written informed consent to participate in this study.

## Author contributions

BD and LC: conceptualization. BD, SJ, and YL: methodology. BD, SJ, YL, HL, RY, NY, CZ, and HW: formal analysis and investigation. BD: writing—original draft preparation. BD, SJ, HL, and ZL: writing—review and editing. LC: funding acquisition and resources. YT and AP: supervision. All authors contributed to the article and approved the submitted version.
