# Supplementary material for: Connection between right-to-left shunt and photosensitivity: a community-based cross-sectional study
Source: Front Neurol. 2023 Apr 27;14:1177879. doi: 10.3389/fneur.2023.1177879 (PMC10172477; doi:10.3389/fneur.2023.1177879)
Supplement: Supplementary file 1 [file Table_1.DOCX]

Supplementary Material

Connection between right-to-left shunt and photosensitivity: a community-based cross-sectional study

Bosi Dong, Shuming Ji, Yajiao Li, Hua Li, Ruiqi Yang, Na Yang, Zhu Liu, Chenxing Zhu, Hui Wang, Yusha Tang, Anjiao Peng, Lei Chen^*^

*** Correspondence:** Lei Chen: leilei_25@126.com

# Supplementary Tables

|  | Yes/No |
| --- | --- |
| 1. I prefer winter over summer because summer lightness bothers me |  |
| 1. If I could, I would prefer to go out after dusk rather than during the day |  |
| 1. I always wear sunglasses when driving or sitting in the car. |  |
| 1. I prefer outdoor activity to indoors |  |
| 1. I wish I could go to the city in the north of China because of short sunshine time |  |
| 1. My ideal house has large windows |  |
| 1. I like cloudy days |  |
| 1. Sunlight is so annoying to me that I have to wear sunglasses when I go out |  |
| 1. I prefer to stay at home on sunny days, even if the weather is not too hot |  |
| 1. I feel reborn in spring when the days start to become longer |  |
| 1. Strong sunlight bothers me |  |
| 1. I prefer rooms that are in semi-darkness |  |
| 1. I have no problem staying on the square or no shelter places in sunny days |  |
| 1. As for color, I prefer yellow than green |  |
| 1. I prefer sunlight to semi-darkness |  |
| 1. Looking at a very bright view annoys me |  |
| 1. If I go to the seaside, I prefer to lie on a rock instead of hiding under the sun umbrella |  |
| 1. I can't stand the light reflecting off the snow |  |
| 1. I think summer make me excited because of the brightness |  |
| 1. I feel uncomfortable when I go directly from dark to bright |  |
| 1. I like spend holidays on seaside since sunlight is like therapy for me |  |
| 1. I always prefer walking in the sunlight unless the temperature is too high |  |
| 1. I do not like bright colors |  |

**Supplementary table 1.** Photosensitivity Assessment Questionnaire**.** Items related to light avoidance behaviors (“photophobia”) were described in 1, 2, 3, 5, 7, 8, 9, 11, 12, 16, 18, 20, 23; meanwhile, items related to related to light-searching behaviors (“photophilia”) were described in 4, 6, 10, 13, 14, 15, 17, 19, 21, 22. Each item is scored zero if answered negatively and 1 if answered affirmatively.

|  | Migraine without aura (n=57) | Migraine with aura (n=4) | Chronic migraine (n=9) | Migraine With Medication Overuse (n=1) |
| --- | --- | --- | --- | --- |
| Gender (male, %) | 9 (15.79) | 1 (25.00) | 3 (33.33) | 1 (—) |
| Age (y, mean±SD) | 42.35 (9.73) | 45.00 (11.25) | 31.56 (9.88) | 26.03 (—) |
| Education (< or = 9 years, %) | 30 (52.63) | 3 (75.00) | 4 (44.44) | 0 (—) |
| BMI (mean±SD) | 22.89 (4.05) | 21.62 (0.99) | 23.67 (2.15) | 25.47 (—) |
| Smoke (n, %) | 7 (12.28) | 0 (0.00) | 1 (11.11) | 0 (—) |
| Alcohol (n, %) | 13 (22.81) | 1 (25.00) | 2 (22.22) | 1 (—) |
| Coffee (n, %) | 11 (19.30) | 1 (25.00) | 3 (33.33) | 1 (—) |
| PHQ9 score (mean±SD) | 2.82 (3.36) | 1.25 (1.30) | 3.11 (3.00) | 1 (—) |
| GAD7 score (mean±SD) | 2.65 (3.71) | 0.00 (0.00) | 0.22 (0.42) | 0 (—) |
| Significant shunt (n, %) | 21 (36.84) | 3 (75.00) | 2 (22.22) | 0 (—) |
| Photosensitivity score (mean±SD) | 5.30 (2.47) | 6.50 (1.66) | 5.44 (3.13) | 8.00 (—) |
| Age at onset (y, mean±SD) | 23.88 (7.65) | 31.50 (14.03) | 17.56 (7.04) | 14.00 (—) |
| Years lived with headache (y, mean±SD) | 18.47 (10.14) | 13.50 (9.01) | 14.00 (10.95) | 12.03 (—) |
| HIT6 (mean±SD) | 54.70 (12.44) | 48.00 (8.34) | 64.44 (3.65) | 66.00 (—) |

**Supplementary table 2.** Characteristics of different types of migraine

|  | Coeffecient (95% CI) of univariate analysis | | | |  |
| --- | --- | --- | --- | --- | --- |
|  | All participants | Healthy controls | | Migraineurs | |
| Significant shunt | 1.362^***^ (1.013, 1.712) | 0.698^**^ (0.262, 1.133) | | 1.654^**^ (0.576, 2.732) | |
| Migraine | 0.711^***^ (0.370, 1.052) | / | | / | |
| Male | -0.153 (-0.612, 0.307) | 0.009 (-0.464, 0.482) | | -0.505 (-2.137, 1.127) | |
| Age | 0.041^**^ (0.015, 0.067) | 0.063^***^ (0.033, 0.093) | | 0.017 (-0.067, 0.101) | |
| BMI | 0.125^***^ (0.068, 0.182) | 0.111^***^ (0.052, 0.170) | | 0.138 (-0.065, 0.341) | |
| Education | -1.151^***^ (-1.551, -0.750) | -0.848^***^ (-1.278, -0.417) | | -1.402^*^ (-2.753, -0.051) | |
| Coffee | 0.612 (-0.407, 0.691) | -0.446 (-1.071, 0.179) | | 0.547 (-1.204, 2.298) | |
| Smoke | -0.196 (-0.812, 0.421) | -0.503 (-1.082, 0.075) | | 0.856 (-1.683, 3.395) | |
| Alcohol | 0.313 (-0.073, 0.698) | -0.235 (-0.656, 0.186) | | 0.825 (-0.439, 2.090) | |
| PHQ9 score | 0.022 (-0.082, 0.127) | -0.005 (-0.127, 0.118) | | 0.030 (-0.297, 0.358) | |
| GAD7 score | 0.046 (-0.047, 0.138) | -0.076 (-0.187, 0.034) | | 0.129 (-0.157, 0.415) | |
| Migraine characteristics | | |  | |  |
| Age at onset | / | / | | 0.050 (-0.005, 0.106) | |
| Years lived with headache | / | / | | -0.037 (-0.089, 0.015) | |
| Aura | / | / | | 1.004 (-0.817, 2.825) | |
| Headache frequency | / | / | | -0.111 (-0.231, 0.009) | |
| Chronic migraine | / | / | | -2.572^*^ (-4.816, -0.330) | |
| HIT6 | / | / | | -0.039 (-0.084, 0.006) | |

*p < .05,

**p < .01,

***p < .001.

**Supplementary table 3.** Univariate analysis of photosensitivity scores in healthy controls and migraineurs.
